# Emergence of a novel genotype of extensively drug-resistant *Shigella sonnei* carrying *blaDHA-1* in diverse patient populations, Los Angeles, 2024–2025

**DOI:** 10.1017/ash.2025.10280

**Published:** 2026-01-13

**Authors:** Shekina Gonzalez-Ferrer, Bennett Shaw, Lilian Fung, Raphael J. Landovitz, Daniel Z. Uslan, Shangxin Yang

**Affiliations:** 1 Department of Pathology and Laboratory Medicine, David Geffen School of Medicine, University of California, Los Angeles, CA, USA; 2 Division of Infectious Diseases, David Geffen School of Medicine, University of California, Los Angeles, CA, USA

## Abstract

Extensively drug-resistant (XDR) *Shigella* is a public health threat, historically linked to *blaCTX-M-15* gene in sexually transmitted cases. We describe a novel XDR *S. sonnei* carrying an AmpC-type *blaDHA-1* gene from two unrelated cases with unknown transmission route; whole-genome sequencing revealed close genetic relatedness, signaling broader community spread.

## Introduction

*Shigella sonnei* is a gram-negative bacterium typically known to cause bacterial gastroenteritis and is one of the causative agents of Shigellosis.^1^ *S. sonnei* is of increasing public health concern due to the extremely drug-resistant (XDR) profile emerging in many strains and is now bypassing *S. flexneri* in lower- or middle-income countries.^2^ In the United States, cases of XDR S. *sonnei* are typically community acquired from men who have sex with men (MSM) populations in metropolitan areas.^3,4^ Nonetheless, there has been an increased frequency in reports of its acquisition in high-risk populations such as unhoused or immunocompromised individuals, including individuals living with HIV, children, and international travelers.^5^ The rise and dissemination of XDR *S. sonnei* has also been observed globally, typically with those carrying extended-spectrum beta-lactamases (ESBL) genes such as *bla*_*CTX-M*_.^5^ These antimicrobial resistance (AMR) genes are often carried on mobile genetic elements, particularly the IncF-like plasmids.^3,5–7^ In our previous studies, we revealed a unique strain of XDR *Shigella sonnei* carrying ESBL-type *bla*_*CTX-M*-15_ spreading among the MSM populations in Los Angeles. As our genomic surveillance continued, we discovered another novel XDR *Shigella sonnei* strain carrying an unusual AmpC-type gene *bla*_*DHA-1*_ in two unrelated cases, one of which had no MSM history.

### Cases

Patient 1 is a 33-year-old immunocompromised female presented to our institution in 2024 with fever (37.2–38.3°C), intermittent chills, rigors, tachycardia, abdominal pain, and diarrhea in the setting of neutropenia. She was diagnosed with high-grade B-cell lymphoma in 2021 and received a bone marrow transplant in 2024. She also had a history of invasive fungal pneumonia. She was thus admitted for neutropenic fever and given meropenem as empiric therapy. She was discharged 7 days later with no recurring diarrhea.

Patient 2 is a 34-year-old male with a history of advanced HIV (CD4 cell count 23 c/mL, viral load 377,000 copies/mL), polysubstance use, and severe mental health disorder presented to our ED with abdominal pain and chronic diarrhea for approximately 6 months. His prior stool studies variably demonstrated *Shigella* spp., for which he was given at various intervals, ceftriaxone monotherapy and levofloxacin monotherapy prior to identifying an XDR *Shigella*. He had a prolonged history of unstable housing and active methamphetamine use and was previously noted as being sexually active with male partners, without more recent exposure history being available. The patient left per patient-directed discharge after four days of intravenous ertapenem.

### Genomic characterization

*S. sonnei* cases were passively identified in our institution first by positive *Shigella* PCR results from the BD Max Enteric Bacterial Panel on stools from patients with gastrointestinal symptoms, followed by stool culture and serotyping. Criteria for XDR *Shigella* were based on the CDC’s guidelines as being resistant to azithromycin, ciprofloxacin, ceftriaxone, trimethoprim-sulfamethoxazole, and ampicillin. Whole-genome sequencing (WGS) of the isolates was performed using the Illumina MiSeq platform. Reference genomes that are most closely related to the isolates were determined by KmerFinder (Center for Genomic Epidemiology, https://cge.cbs.dtu.dk/services). Isolates were further genotyped by using Pathogenwatch web application (https://pathogen.watch/, accessed 3/30/25). Qiagen CLC Genomics Workbench (v23.0.4) was used for single nucleotide polymorphism (SNP) analysis. More than 0.5 million reads were acquired for each isolate, ensuring the mean whole genome coverage to be at least 25X, as previously reported.^8^ Center for Genomic Epidemiology tools were used, including CSI Phylogeny 1.4 for SNP calling inferring Phylogeny, ResFinder for AMR gene identification, and PlasmidFinder for plasmid replicon identification. The isolates from Cases 1 and 2 were identified as S. sonnei ST152, and were most closely related to the reference strain 506 isolated from a stool specimen, from a women’s hospital in Boston, United States (NZ_CP053751.1) with a ∼99% genome coverage and pairwise identity.

**Figure 1.**
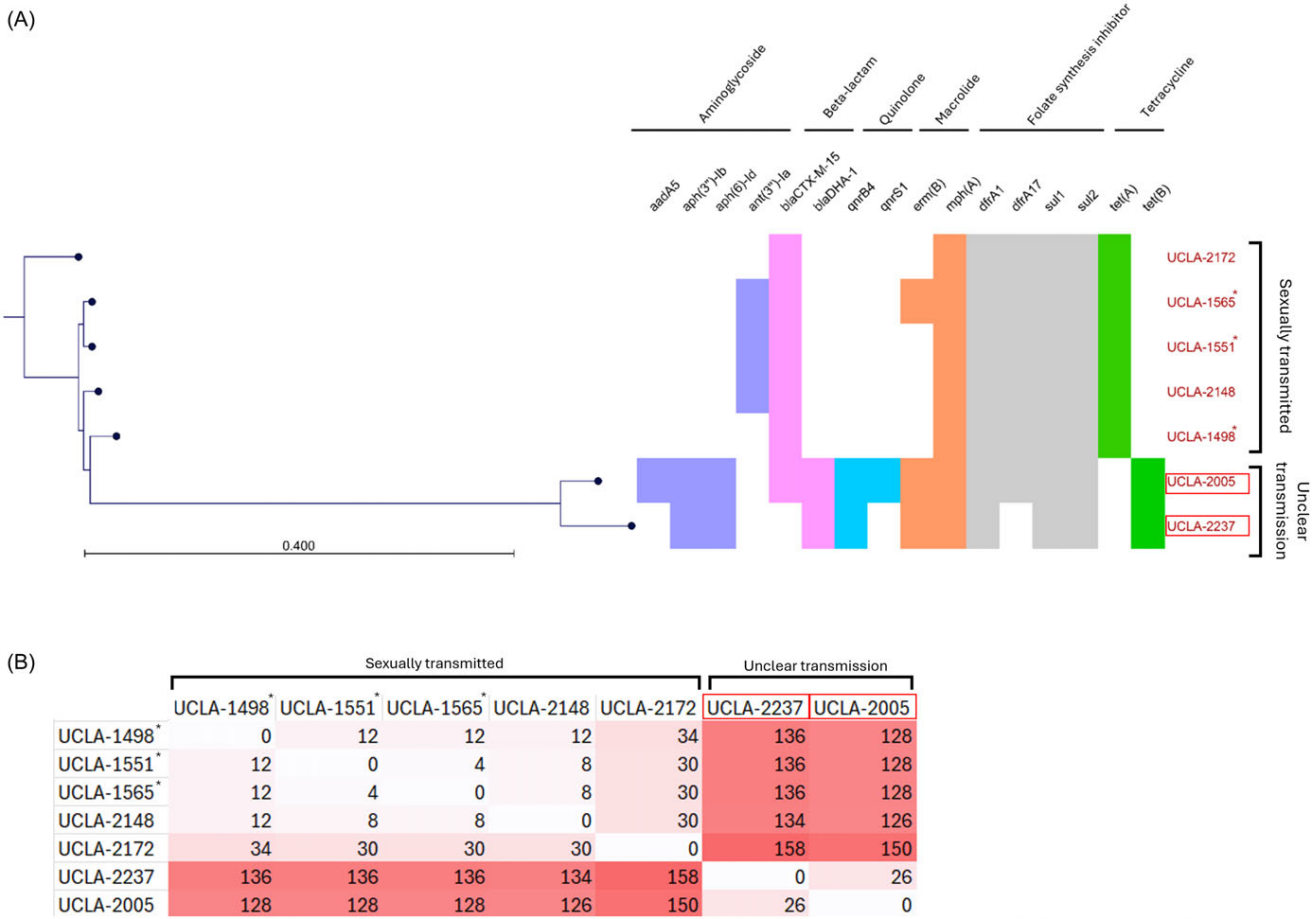
Genotypic characterization of *Shigella sonnei* isolates. A) Phylogenetic tree showing clustering of the two *blaDHA-1*-carrying XDR *Shigella sonnei* isolates and AMR genes across *Shigella sonnei* isolates. UCLA isolates 1,498*, 1551*, and 1565* have been previously published. B) SNP matrix showing number of SNP differences between UCLA isolates.

Both isolates exhibited very similar resistance profiles (Table 1). Both isolates were resistant to oral drugs commonly used to treat shigellosis, including azithromycin, ciprofloxacin, and trimethoprim/sulfamethoxazole, attributing to AMR genes conferring resistance to macrolides (*erm*(B), *mph*A), quinolones (*qnrB4, qnrS1*), sulfonamides (*sul1, sul2*), and trimethoprim (*dfrA1, dfrA17*). Both were resistant to ampicillin, amoxicillin/clavulanate, and third-generation cephalosporins but susceptible to piperacillin/tazobactam and carbapenems. Most notably, Case 1 (UCLA 2005) carried both an ESBL-type *bla*_*CTX-M-15*_ and an AmpC-type *bla*_*DHA-1*_, while Case 2 (UCLA 2,237) only carried *bla*_*DHA-1*_ (Figure 1). Despite both having tet(B) gene, predicting resistance to minocycline, UCLA 2005 was intermediate (MIC = 8) while UCLA 2,237 was susceptible (MIC = 2). Additionally, 3 AMR genes conferring resistance to aminoglycosides were detected in both isolates including *aadA5, aph(3″)-Ib, and aph(6)-Id*, however, this class was phenotypically susceptible. These discrepancies between the genotype and phenotype require further investigation. Possible reasons include insufficient AMR gene expression and low-level resistance that’s below the limit of detection of the phenotypic antibiotic susceptibility test.

**Table 1.** Antimicrobial susceptibility profile for XDR *Shigella sonnei* isolates

	Case 1 (UCLA 2005)		Case 2 (UCLA 2,237)	
Antibiotic	MIC	Interpretation	MIC	Interpretation
Amikacin	≤4	S	≤4	S
Amoxicillin/Clavulanate	>32	R	>32	R
Ampicillin	>32	R	>32	R
Cefazolin	>32	R	>32	R
Cefepime	2	S	≤.5	S
Ceftazidime	8	I	32	R
Ceftazidime/Avibactam	≤2	S	≤2	S
Ceftriaxone	>64	R	4	R
Ciprofloxacin	>4	R	>4	R
Ertapenem	≤.25	S	≤.25	S
Gentamicin	2	S	≤1	S
Imipenem	≤1	S	≤1	S
Levofloxacin	>8	R	8	R
Meropenem	≤.25	S	≤.25	S
Minocycline	8	I	2	S
Piperacillin/Tazobactam	≤8	S	≤8	S
Tigecycline	1	S	≤.25	S
Tobramycin	≤1	S	≤1	S
Trimethoprim/Sulfamethoxazole	>4/80	R	>4/80	R

MIC = minimal inhibitory concentration, S = susceptible, I = Intermediate, R = Resistant.

To investigate the genetic relatedness of the isolates from Cases 1 and 2 (UCLA 2005 and UCLA 2,237), we compared them with five sexually transmitted XDR *S. sonnei* isolates with clear epidemiologic link to MSM, including three from prior reports (UCLA 1,498*, 1551*, 1565*)^3^ and two additional unpublished isolates (UCLA 2,148, 2,172). While all isolates analyzed in this study were identified as lineage 3.6.1.1 using mykrobe v0.13.0^9^, phylogenetic analysis showed 2 distinctive clusters of XDR *S. sonnei* (differed by 100 SNPs), with the sexually transmitted isolates all carrying *bla*_*CTX-M-15*_ and tet(A), while the isolates with unclear transmission route all carrying *bla*_*DHA-1*_ and tet(B) (Figure 1). Further, despite no known epidemiologic link between Cases 1 and 2, the two isolates (UCLA 2005 and UCLA 2,237) only had 26 SNP differences, suggesting a relatively close relatedness and a recent common ancestor strain. In addition, both isolates harbored a IncFII-like plasmid that was closely related to *bla*_*DHA-1*_ carrying plasmids found in a *S. flexnerii* isolated from a non-MSM patient in Australia (CP061363.1),^10^ and a *S. sonnei* isolated from a patient in Taiwan (CP151315.1).

## Discussion

This is the first report describing a *bla*_*DHA-1*-_carrying XDR *Shigella* in the U.S. The most concerning finding was that this novel strain was found in patients with unclear transmission route, highlighting the wider spread and continued evolution of XDR *Shigella* in the community. Strikingly, both *bla*_*CTX-M-15*_ and *bla*_*DHA-1*_ genes were found in the XDR *Shigella* in a non-MSM patient without a significant travel history or other risk factors. This genotype has never been reported before.

These two isolates, discovered one year apart, differed by only 26 SNPs, suggesting a close genetic relationship and supporting the hypothesis of community transmission of another unique strain of XDR *Shigella*. There are no identifiable epidemiological links between the two patients, who resided 80 miles from each other and did not overlap at our institution in either location or time. Additionally, there were no known unusual food exposures in either patient or an awareness for an outbreak of foodborne *Shigella* during the time of these cases.

While previous reports have described *Shigella sonnei* carrying either AmpC or ESBL genes individually,^6,11–14^ none have demonstrated co-carriage of both *bla*_*CTX-M-15*_ and *bla*_*DHA-1*_. Other countries have reported co-carriage of *bla_CTX-M-15_* and AmpC (*bla_CMY-2_* or *bla_CIT_*) genes in *S. sonnei* isolates co-resistant to 3^rd^ gen cephalosporins and fluoroquinolones.^15–19^ Other Enterobacterales such as *E. coli* and *K. pnuemoniae* carrying plasmid-borne AmpC and ESBLs have been documented.^20^ *K. pneuomoniae* have been reported to co-carry *bla*_*DHA-1*_ and *bla*_*CTX-M-3*_.^21^ What is particularly concerning here is the emergence of *bla*_*DHA-1*_ on a plasmid in a community-acquired *Shigella* isolate, suggesting possible horizontal gene transfer between species within Enterobacterales. The clinical implications of this are significant given that dual cephalosporinase producers may exhibit higher resistance than the more typical CTX-M–associated XDR *Shigella*. While global spread of XDR *Shigella* phenotypes have been more attributed to CTX-M carriage,^22^ the emergence of DHA-1-carrying XDR *Shigella* in more diverse patient populations signaled a different epidemiologic dynamic and warrant close monitoring and continuous genomic surveillance.

## Data Availability

The WGS sequences can be found via NCBI Genbank (PRJNA1335817 and PRJNA1085759).
